# Structural and functional analysis of two small leucine-rich repeat proteoglycans, fibromodulin and chondroadherin

**DOI:** 10.1016/j.matbio.2017.02.002

**Published:** 2017-11

**Authors:** Patricia Paracuellos, Sebastian Kalamajski, Arkadiusz Bonna, Dominique Bihan, Richard W. Farndale, Erhard Hohenester

**Affiliations:** a Department of Life Sciences, Imperial College London, London, United Kingdom; b Department of Medical Biochemistry and Microbiology, Uppsala University, Sweden; c Department of Biochemistry, University of Cambridge, Cambridge, United Kingdom

**Keywords:** Leucine-rich repeat, Collagen, X-ray crystallography

## Abstract

The small leucine-rich proteoglycans (SLRPs) are important regulators of extracellular matrix assembly and cell signalling. We have determined crystal structures at ~ 2.2 Å resolution of human fibromodulin and chondroadherin, two collagen-binding SLRPs. Their overall fold is similar to that of the prototypical SLRP, decorin, but unlike decorin neither fibromodulin nor chondroadherin forms a stable dimer. A previously identified binding site for integrin α2β1 maps to an α-helix in the C-terminal cap region of chondroadherin. Interrogation of the Collagen Toolkits revealed a unique binding site for chondroadherin in collagen II, and no binding to collagen III. A triple-helical peptide containing the sequence GAOGPSGFQGLOGPOGPO (O is hydroxyproline) forms a stable complex with chondroadherin in solution. In fibrillar collagen I and II, this sequence is aligned with the collagen cross-linking site KGHR, suggesting a role for chondroadherin in cross-linking.

## Highlights

• The crystal structures of fibromodulin and chondroadherin have been determined.
• Fibromodulin and chondroadherin are monomeric in solution.
• Chondroadherin binds to a unique site in type II collagen that contains the sequence GAOGPSGFQGLOGPOGPO (O, hydroxyproline).
• In collagen fibres, the chondroadherin binding site is adjacent to the cross-linking site, KGHR.

## Introduction

The small leucine-rich proteoglycans (SLRPs) constitute a family of secreted proteins with important roles in extracellular matrix assembly [1], [2] and cell signalling [3]. SLRPs are divided into five classes based on sequence homology. Despite their name, not all SLRPs are modified with glycosaminoglycan chains, and several of them are additionally modified by tyrosine sulphation [1], [2]. The biological functions of SLRPs have been studied in knockout mice and the findings so far suggest tissue-specific functions of each SLRP, conveyed through defined temporospatial expression patterns and interactions with collagen. The data also point to differential roles of SLRPs in organising collagens into tissue-specific supramolecular structures [1], [2].

Fibromodulin, a class II SLRP, is associated with dense collagen matrix in tendons and ligaments, as well as in fibrotic tissues, tumors and atherosclerotic plaques [4], [5], [6]. In fibromodulin-deficient mouse tendons, collagen fibrillogenesis is dysregulated: collagen fibrils are misassembled [7], collagen α chains are aberrantly cross-linked, and collagen C-telopeptides are excessively oxidised by the collagen cross-linking enzyme, lysyl oxidase [8]. A possible mechanism for fibromodulin's role in collagen fibrillogenesis is its recruitment to collagen cross-linking sites and also its interaction with, and apparent effect on, lysyl oxidase [9].

One of the more distant homologues of fibromodulin is chondroadherin, a class IV SLRP found in cartilage and bone [10]. Chondroadherin-deficient mice have thinner cortical bones and longer growth plate proliferation zones [11], as well as mechanically softer knee surface cartilage [12]. Chondroadherin interacts with collagen II [13], and mediates cell-matrix interactions through binding to integrin α2β1 [14] and heparan sulphate [15].

The SLRPs belong to the large superfamily of leucine-rich repeat (LRR) proteins [16], [17], which includes not only secreted proteins but also a large number of cell surface receptors, such as the Toll-like receptors involved in innate immunity [18], the platelet von Willebrand factor receptor, glycoprotein Ib [19], [20], and proteins involved in neural development [21], [22]. The defining feature of these proteins is the presence of multiple repeats of 20–30 amino acids in length and starting with the consensus sequence LxxLxLxxNxL (L can be substituted by I, V or other hydrophobic residues). The folding principle of LRR proteins was revealed by the crystal structure of ribonuclease inhibitor [23]: the conserved leucine residues form the hydrophobic core of a curved solenoid structure that is characterised by an inner concave face composed of parallel β-strands and an outer convex face composed of variable structure. In proteins with long LRRs, such as ribonuclease inhibitor, the outer face consists of α-helices and the solenoid is highly curved; in proteins with short LRRs, the helices are replaced by loops and turns, which reduces the solenoid's curvature [16].

Interaction partners of LRR proteins frequently bind to the concave face [16]. Indeed, several modelling and mutational studies have implicated the concave face of SLRPs in collagen binding [4], [24], [25], [26], [27], [28], [29]. The crystal structure of the prototypical class I SLRP, decorin, revealed a tight dimer in which most of the concave face is occluded [30]. This finding led to a controversy about the physiological state of decorin and other SLRPs [31], [32], [33], which was only recently resolved by the demonstration that decorin dimerisation is reversible, allowing the monomer to bind collagen through its concave face [26]. Even so, further structural studies of the SLRP family are clearly warranted. At the time of writing, the only crystal structures available were those of two closely related class I SLPRs, decorin [30] and biglycan [33]; a chondroadherin structure was published while this manuscript was in preparation [34]. Here, we report the crystal structure of fibromodulin and a different crystal form of chondroadherin, which reveal that these SLRPs do not self-associate in the same manner as decorin or biglycan. We also identify, using the Collagen Toolkits [35], a unique collagen sequence that binds with high affinity to chondroadherin and is distinct from sequences that bind fibromodulin.

## Results

### Crystal structure of fibromodulin

The LRR region of fibromodulin is preceded by ~ 50 residues predicted to be unstructured and including a number of sulphated tyrosines [36]. We obtained crystals of a natively glycosylated human fibromodulin construct in which ten N-terminal tyrosines were mutated to serine to abolish sulphation (N-terminally truncated fibromodulin was not well expressed). The fibromodulin crystal structure at 2.21 Å resolution (Table 1) is complete except for the disordered N-terminal region. The asymmetric unit of the crystals contains two structurally very similar copies of fibromodulin (r.m.s. deviation of 0.2 Å for 306 Cα atoms). Human fibromodulin contains four potential *N*-linked glycosylation sites at Asn127, Asn166, Asn201, and Asn291. We observed electron density for the innermost four sugar moieties at all four sites, namely the two *N*-acetyl-glucosamines of the chitobiose core, the branching mannose, and a fucose α1,6-linked to the first *N*-acetylglucosamine. Whether any of these glycans is extended by keratan sulphate [37], [38] could not be determined from the electron density.

**Table 1:** Crystallographic statistics.

	Fibromodulin	Chondroadherin
*Data collection*		
Beamline	I04-1	I24
Wavelength (Å)	0.9282	1.739
Resolution range (Å)	65.9–2.21 (2.27–2.21)	58.4–2.17 (2.23–2.17)
Space group	*C*2	*C*2
Unit cell dimensions		
*a*, *b*, *c* (Å)	108.11, 98.93, 111.34	215.45, 60.70, 57.66
α, β, γ (°)	90, 107.39, 90	90, 100.60, 90
Unique reflections	55,434	37,325
Multiplicity	6.8 (6.9)	5.8 (3.9)
Completeness (%)	99.0 (99.1)	95.8 (75.3)
Mean I/σ(I)	9.5 (1.1)	9.3 (1.6)
CC_1/2_	0.998 (0.523)	0.987 (0.506)
R_merge_	0.111 (1.81)	0.148 (0.840)
*Refinement*		
Protein atoms	5268	5210
Solvent atoms	566 H_2_O, 2 Ni^2 +^, 2 Cl^−^, 1 SO_4_^2 −^	412 H_2_O, 2 Ni^2 +^, 1 Cl^−^, 3 PO_4_^3 −^
R_work_	0.179	0.188
R_free_	0.216	0.240
R.m.s.d. bonds (Å)	0.003	0.003
R.m.s.d. angles (°)	0.76	0.69
Ramachandran plot		
Favoured (%)	95.1	92.8
Outliers (%)	0	0

**Fig. 1:**
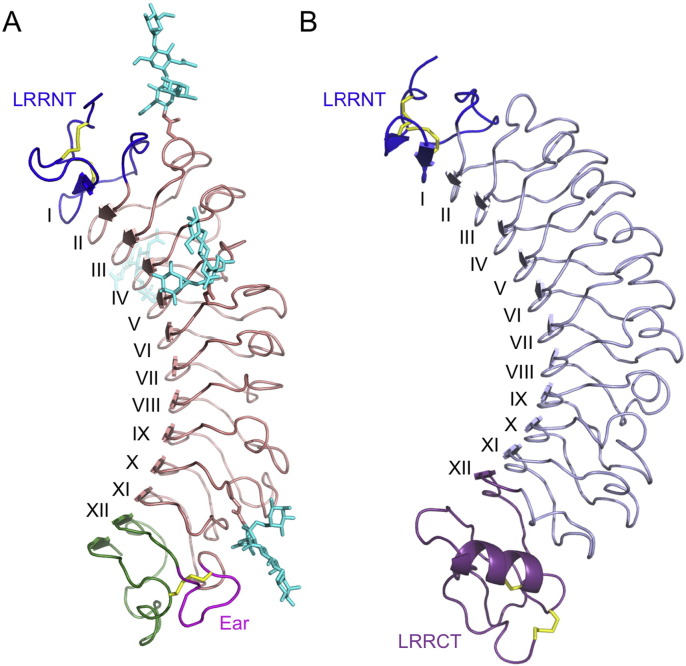
Crystal structures of (A) fibromodulin and (B) chondroadherin. The LRRNTs are coloured blue in both proteins and disulphide bonds are shown as yellow sticks. The C-terminal cap of fibromodulin is in green, with the ear loop highlighted in magenta. The four *N*-linked glycans of fibromodulin are shown as cyan sticks. The LRRCT of chondroadherin is in purple. The LRRs are labelled with roman numerals (see Fig. S1 for sequences). Crystal structures of (A) fibromodulin and (B) chondroadherin. The LRRNTs are coloured blue in both proteins and disulphide bonds are shown as yellow sticks. The C-terminal cap of fibromodulin is in green, with the ear loop highlighted in magenta. The four *N*-linked glycans of fibromodulin are shown as cyan sticks. The LRRCT of chondroadherin is in purple. The LRRs are labelled with roman numerals (see Fig. S1 for sequences).

Fibromodulin adopts the curved solenoid structure characteristic of all LRR proteins (Fig. 1A). The N-terminal cap (LRRNT, 79-106) contains two conserved disulphide bonds (Cys76-Cys82, Cys80-Cys92) and seals the hydrophobic core of the LRR region. The cap contributes one strand to the curved parallel β-sheet that dominates the concave face of fibromodulin. In the decorin crystal structure, this strand was designated as the first LRR [30]. In fibromodulin, its sequence deviates substantially from the LRR consensus, but we retain the decorin numbering for consistency. The LRRNT of fibromodulin is followed by eleven LRRs (II-XII) that contain the consensus sequence LxxLxLxxNxL. The total length of the LRRs varies between 20 and 27 residues (Fig. S1A). The LRRs in fibromodulin follow a long-long-short pattern, as previously described for decorin [30]. Residues 3–6 of each LRR contribute one β-strand to the concave face of the curved solenoid; the convex face is made up of loops and turns. LRR XI contains a protruding loop that is characteristic of SLRP classes I, II or III and has been termed the “ear” [30]. The ear spans from the first conserved C-terminal cysteine (Cys334, disulphide bonded to Cys367) to the β-strand of LRR XII. The last five residues of fibromodulin form the 13th strand of the concave β-sheet.

**Fig. 2:**
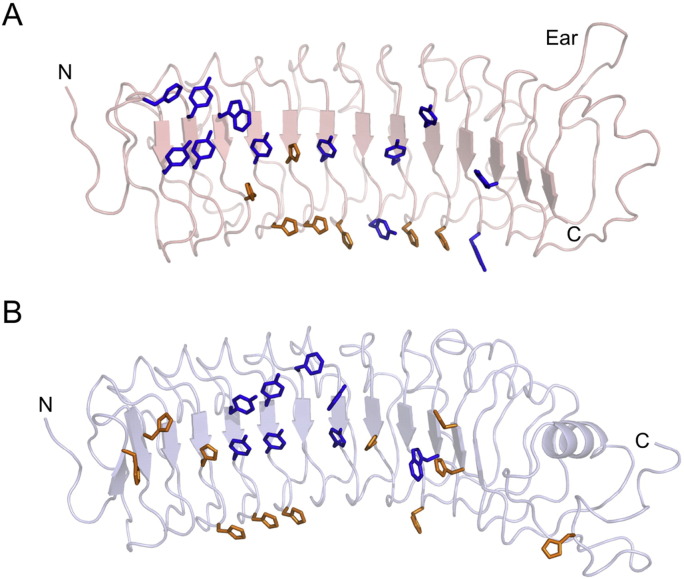
Histidines and aromatic residues on the concave faces of (A) fibromodulin and (B) chondroadherin. Histidines are shown in orange, aromatic residues (Phe, Tyr, Trp) are shown in blue. The N- and C-termini are labelled.

**Fig. 3:**
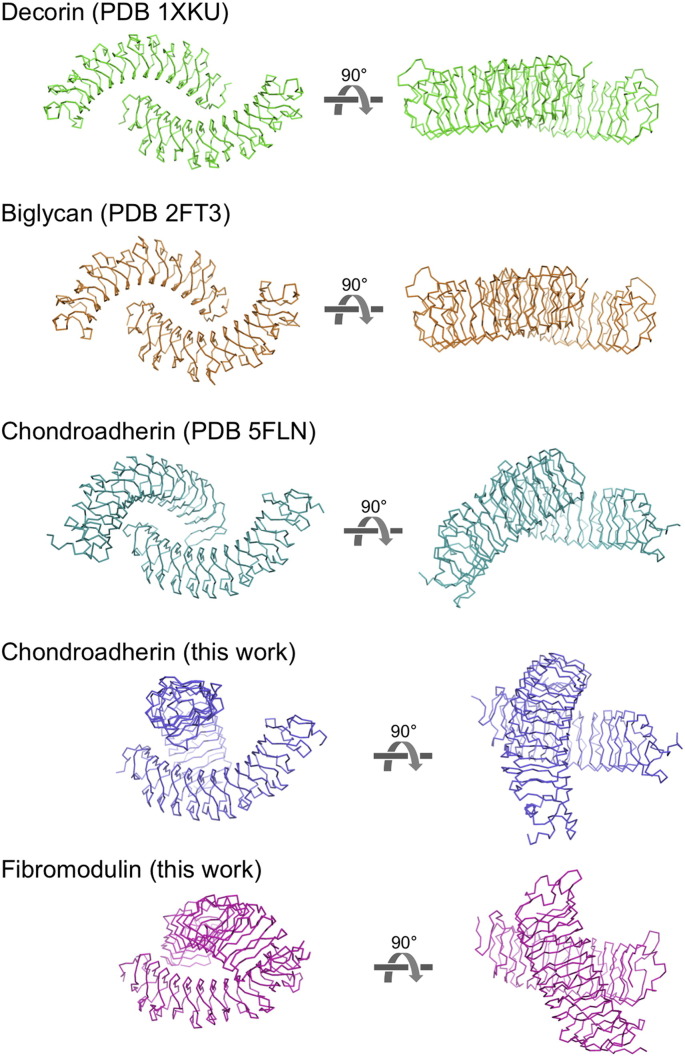
Concave face interactions in SLRP crystal structures. For each structure, two orthogonal views are shown. Decorin and biglycan form stable dimers in solution [26], [32], [33]. In contrast, fibromodulin and chondroadherin are predominantly monomeric in solution (this work, Fig. 4) and the interactions shown are likely to be crystal lattice artefacts.

**Fig. 4:**
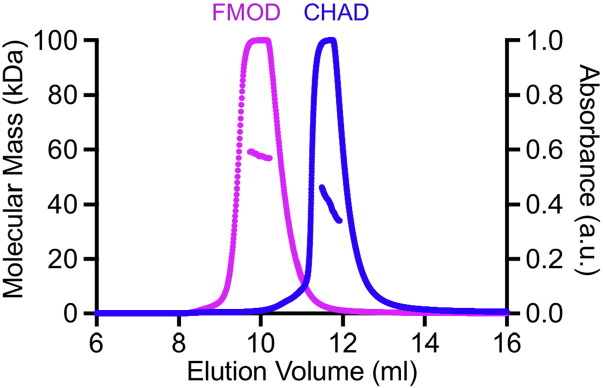
Oligomeric states of fibromodulin and chondroadherin in solution. The proteins were injected onto a size exlusion column at a concentration of 10 mg/ml and the molecular masses determined by multi-angle light scattering. The absorbance peaks are truncated due to saturation of the signal. FMOD, fibromodulin; CHAD, chondroadherin.

**Fig. 5:**
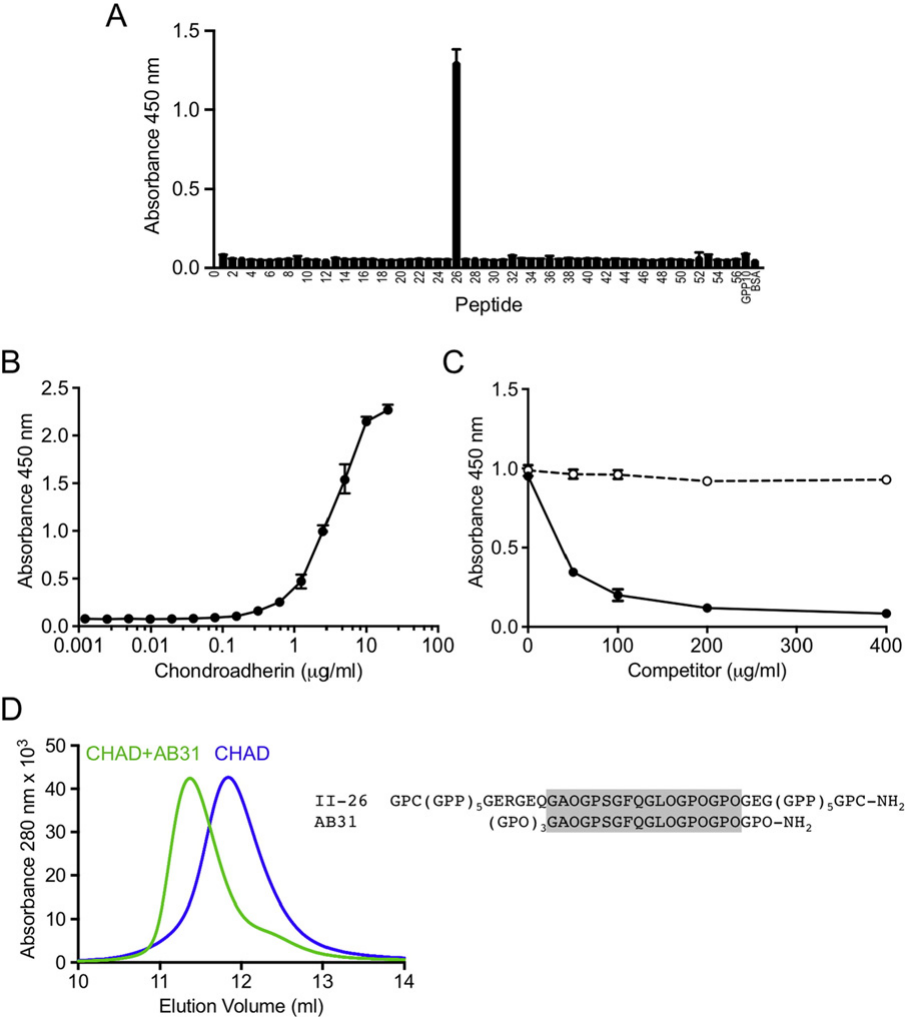
Collagen binding by chondroadherin. (A) Binding of biotinylated chondroadherin to immobilised Collagen Toolkit II peptides. Binding was detected with streptavidin-HRP and TMB substrate (absorbance at 450 nm). Error bars show standard deviation from three technical replicates. (B) Dose-response curve of chondroadherin binding to peptide II-26 peptide interaction. (C) Inhibition of the chondroadherin-collagen II interaction by peptide II-26 peptide (solid line) or peptide III-8 (negative control, dashed line). (D) Binding of peptide AB31 to chondroadherin (CHAD) in solution analysed by size exclusion chromatography. AB31 and chondroadherin were mixed in a 2:1 molar ration and incubated for 15 min before injection onto the Superdex 75 column. The sequences of peptides II-26 and AB31 are indicated on the right and the sequence common to both is shaded.

**Fig. 6:**
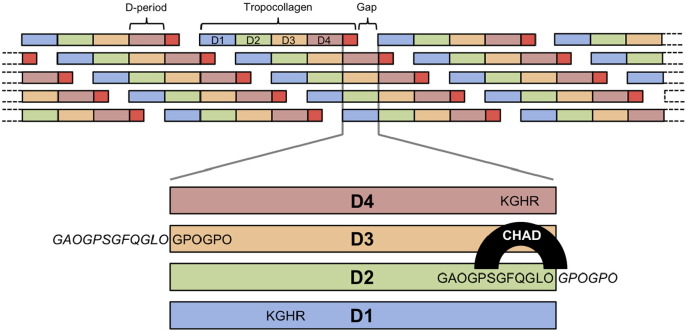
Alignment of chondroadherin binding and cross-linking sites in collagen fibres. Shown at the top is the staggered arrangement of tropocollagen molecules within a collagen fibre, with D-periods labelled D1 to D4 (the red segment is D5). The expanded view shows the vertical alignment of D1–D4. The chondroadherin-binding sequence from Toolkit peptide II-26 is located mainly in D2, but extends into D3 (sequences from the adjacent D-period are in italics). The KGHR cross-linking motif in D4 aligns exactly with the chondroadherin binding site in D2. The KGHR motif in D1 is axially displaced by ~ 40 nm towards the N-terminus of the fibre.

### Crystal structure of chondroadherin

In contrast to fibromodulin, chondroadherin does not contain an N-terminal tyrosine sulphate-rich region or any *N*-linked glycans. We obtained crystals of human chondroadherin and determined the structure at 2.17 Å resolution (Table 1). The structure is complete except for the C-terminal 13 residues. The asymmetric unit of the crystals contains two structurally similar copies of chondroadherin (r.m.s. deviation of 0.7 Å for 327 Cα atoms).

Chondroadherin also adopts a curved solenoid structure, but there are a number of differences with respect to the fibromodulin structure described above (Fig. 1B). Firstly, the LRRNT domain of chondroadherin more closely corresponds to the consensus structure with two anti parallel β-strands [39], and its second strand is part of a standard LRR motif (Fig. S1B). Secondly, the LRRs II-XI of chondroadherin are more uniform in length (24–25 residues), resulting in a slightly higher curvature of the concave face (Fig. 1). Thirdly, the ear and LRR XII of fibromodulin are replaced by a C-terminal cap (LRRCT) in chondroadherin. The LRRCT is a common C-terminal motif of many LRR proteins that are not SLRPs, such as glycoprotein Ibα [19], [20], Nogo receptor [40], [41], Slit [42] and Toll-like receptors [43]. The LRRCT is characterised by a 10-residue α-helix and two disulphide bonds (Cys304-Cys326 and Cys306-Cys346 in chondroadherin).

Comparison of our chondroadherin structure (crystallised at pH 4.8) with the structure recently published (crystallised at pH ~ 10) [34] reveals no noteworthy differences in tertiary structure: the average r.m.s. deviation of the eight pairwise superpositions of crystallographically independent chains is 0.94 ± 0.31 Å (322 Cα atoms). However, the chondroadherin monomers are packed differently in the two crystal lattices (see below).

### Distribution of histidines and aromatic residues

We noticed that untagged fibromodulin and chondroadherin showed substantial affinity for Ni^2 +^ resin and exploited this property for the purification of chondroadherin. In the crystal lattices of both fibromodulin and chondroadherin, neighbouring molecules are linked by heavy atoms coordinated by multiple histidine residues, most likely Zn^2 +^ or Ni^2 +^ (not shown). These observations prompted us to examine the distribution of histidines in fibromodulin and chondroadherin. We found that histidines, as well as aromatic residues, are markedly concentrated on the concave faces of fibromodulin and chondroadherin (Fig. 2), explaining their binding to Ni^2 +^ affinity resin. Fibromodulin and chondroadherin both have prominent aromatic patches in the N-terminal halves of their concave faces, which are likely sites of protein-protein interactions. Indeed, as described in the next paragraph, these patches mediate important lattice interactions in the crystals.

### Comparison of SLRP crystal structures

Previously, crystal structures of the core proteins of two class I SLRPs were reported: decorin [30] and biglycan [33]. Pairwise superpositions show that decorin and biglycan more closely resemble each other than either fibromodulin or chondroadherin (Table 2). However, there is no straightforward correlation between sequence identity and structural similarity, as measured by the r.m.s. deviation of global superpositions. Apart from chondroadherin uniquely containing a LRRCT, the four SLRP structures differ subtly in the curvature of their β-sheets and in the connecting loops on the convex outside of the curved solenoid.

**Table 2:** Pairwise sequence identities and structural similarities of four SLRPs. The r.m.s. deviations were calculated with the program PDBe Fold v2.59 [59] and the number of matched Cα atoms is indicated in parentheses.

	Fibromodulin	Decorin	Biglycan
Chondroadherin	26% 2.2 Å (267)	26% 1.7 Å (279)	27% 2.0 Å (277)
Fibromodulin		26% 2.1 Å (281)	23% 2.6 Å (269)
Decorin			58% 1.5 Å (301)

The decorin and biglycan structures revealed a conserved mode of dimerisation, consistent with the strong tendency of these proteins to dimerise in solution [30], [32], [33]. The crystal packing of fibromodulin and chondroadherin does not show the same mode of association (Fig. 3). While there are contacts between the concave faces in the fibromodulin and chondroadherin crystals, the paired molecules are arranged differently, with the two solenoids crossing at an angle rather than forming the elongated dimers of decorin and biglycan. Notably, the packing of chondroadherin molecules in our crystal structure is different from that seen in another chondroadherin structure [34], suggesting that the concave face interactions are not physiologically relevant (Fig. 3).

We used size exclusion chromatography with multi-angle light scattering (SEC-MALS) to determine the oligomeric states of fibromodulin and chondroadherin in solution (Fig. 4). The experimentally determined mass of fibromodulin was 56.7 kDa (calculated: 41.5 kDa + 4 *N*-linked glycans) and that of chondroadherin was 39.6 kDa (calculated: 38.6 kDa). In comparison, using the same SEC-MALS setup, we previously determined an experimental mass of 83.5 kDa for the glycosylated decorin core protein (calculated: 36.2 kDa + 4 *N*-linked glycans) [26]. The results demonstrate that, unlike decorin, neither fibromodulin nor chondroadherin forms a stable dimer in solution. Given that the UV absorbance of the eluting peaks was saturated in our SEC-MALS experiment (Fig. 4), we estimate that the peak protein concentration was at least 50 μM. Thus, if fibromodulin or chondroadherin have any tendency to form dimers, their dissociation constant would have to be substantially higher than that of the decorin dimer, which is 1.4 μM [26].

As noted previously [30], [39], the interface residues in the decorin and biglycan dimers are highly conserved (79% identity). These residues, in particular those involved in hydrogen bonds or salt bridges, are poorly conserved in fibromodulin (21%) and chondroadherin (24%). We also note that the residues involved in the dimeric crystal contacts of fibromodulin and chondroadherin are not conserved between these two proteins.

### Identification of a unique chondroadherin binding site in collagen

The Collagen Toolkits are a set of overlapping triple-helical peptides, designed to allow the mapping of collagen-binding proteins onto collagens II and III with 9-residue resolution [35]. Here, Collagen Toolkit II was used in solid-phase binding assays to investigate binding sites for chondroadherin. A single Toolkit peptide, II-26, supported chondroadherin binding; all other peptides showed only basal levels of binding (Fig. 5A). No binding of chondroadherin to Collagen Toolkit III was detected (not shown). Dose-dependent binding of chondroadherin to II-26 reached half-maximum saturation at ~ 3 μg/ml chondroadherin, corresponding to an apparent dissociation constant of ~ 75 nM (Fig. 5B), and II-26 inhibited the binding of chondroadherin to immobilised collagen II (Fig. 5C). We prepared a truncated version of II-26, AB31, for co-crystallisation trials. AB31 formed a complex with chondroadherin in solution (Fig. 5D), demonstrating that the GAOGPSGFQGLOGPOGPO sequence (O is hydroxyproline) shared by II-26 and AB31 provides a high-affinity binding site for chondroadherin. This sequence is located mainly within D-period D2 but extends across the D2-D3 boundary (Fig. 6). Further work is needed to identify exactly those residues that interact with chondroadherin. The purified chondroadherin-AB31 complex crystallised readily at low pH, but unfortunately the crystals invariably contained only chondroadherin and not the peptide. We were unable to obtain crystals at neutral pH, where the chondroadherin-AB31 complex appears to be quite stable (Fig. 5D).

## Discussion

The fibromodulin and chondroadherin structures reported here reinforce the notion that SLRPs are versatile scaffolds for protein interactions. While the overall fold of SLRPs is conserved and can be predicted reliably from homology, their modes of self-association vary and cannot be predicted. Decorin and biglycan were found to form very similar dimers that are quite stable in solution [30], [33]. Fibromodulin and chondroadherin, in contrast, do not dimerise stably in solution, and their crystal packing shows a wide range of, presumably non-physiological, concave face interactions (Fig. 3). A recent study proposed that chondroadherin may form physiological dimers, but this proposal was based solely on an analysis of crystal lattice interactions and computational docking [34]. Our SEC-MALS experiment (Fig. 4) shows that chondroadherin dimerisation in solution is, at best, very weak. We speculate that the frequent self-association of LRR-containing proteins through their concave faces may be driven by general geometric and physico-chemical reasons: for curved solenoid structures, the clasping of their concave surfaces may be the easiest way of burying surface areas that frequently contain exposed aromatic residues.

Previous studies with bacterially expressed fibromodulin fragments identified LRRs 5-7 and 11 (VI-VIII and XII in the numbering scheme used here) as important for collagen binding [4], [25]. Thus, the collagen binding site appears to be located in the C-terminal half of fibromodulin, potentially leaving the N-terminal half containing most of the surface-exposed aromatic residues free to interact with another partner. Lysyl oxidase has been shown to bind to the very N-terminus of fibromodulin [9], which is not ordered in our crystal structure.

The collagen-binding site on chondroadherin has not been mapped. The C-terminal 14 residues of chondroadherin, which are not resolved in our structure, bind heparan sulphate [15]. Chondroadherin residues 307–318 have been identified as a binding site for integrin α2β1 [44]. These positions map to the α-helix in the C-terminal cap of chondroadherin. Glu316, the last residue of the α-helix, appears to be critical for integrin binding and may interact with the metal ion-dependent adhesion site of the integrin α2 I domain [45], [46].

A major finding of the present study is that chondroadherin binds to a unique sequence motif in collagen II, GAOGPSGFQGLOGPOGPO (six of the nine triplets of primary sequence in Toolkit peptide II-26), and not at all to collagen III. In this respect, chondroadherin differs from fibromodulin, which bound several Toolkit peptides from both collagens II and III [9]. The chondroadherin-binding motif has a GFx triplet in common with several other collagen-binding proteins, but only one other protein, OSCAR, has been mapped to peptide II-26 to date [47]. Chondroadherin knockout mice present with a widened epiphyseal growth plate, consistent with impaired transition from cartilage to bone, which may imply a role for chondroadherin in facilitating the osteoclastic resorption of calcified cartilage. II-26 contains a 15-residue sequence that is sufficiently conserved in both the α1 and α2 chains of collagen I, but not in collagen III, to suggest that this same site in collagen I supports the reported binding of chondroadherin to collagen I [11]. It is also of interest that, in the alignment of collagen D-periods (Fig. 6), the II-26 sequence is located mainly within the C-terminal end of D2 where its GFQ triplet aligns perfectly with the collagen cross-linking site, KGHR, in D4 that is the target for condensation with oxidised lysine in nearby N-collagen telopeptides. This suggests a potential role for chondroadherin in collagen fibril assembly and cross-linking, as previously suggested [13], although a later study reported normal organisation of bone collagen in chondroadherin knockout mice but altered mechanical properties and trabecular bone organisation [11]. We have previously proposed a role for fibromodulin in collagen cross-linking, which itself binds to KGHR-containing sites in Collagen Toolkits II and III [9].

## Materials and methods

### Expression vectors

The coding sequence for human fibromodulin with ten tyrosine residues in the N-terminal region mutated to serine (Y38S, Y39S, Y42S, Y45S, Y47S, Y50S, Y53S, Y55S, Y63S, Y65S) was synthesised by Genscript and cloned into a modified pCEP-Pu vector containing the BM-40 signal peptide sequence [48]. The fibromodulin construct comprises residues 19–376 of UniProt Q06828 and contains a tobacco etch virus (TEV) protease-cleavable His-tag at the N-terminus [49]; after secretion and TEV protease treatment, a vector-derived GALA sequence remains present at the N-terminus. The coding sequence for human chondroadherin was amplified from cDNA and cloned into the original pCEP-Pu vector containing the BM-40 signal peptide sequence [48]. The construct comprises residues 23–359 of Uniprot O15335 and is untagged; after secretion, a vector-derived APLA sequence remains present at the N-terminus. All expression vectors were verified by DNA sequencing.

### Protein expression and purification

Human embryonic kidney HEK293 c18 cells (ATCC) were used for protein production. The cells were grown at 37 °C with 5% CO_2_ in Dulbecco's modified Eagle medium F12 (Thermo Fisher Scientific) containing 10% fetal bovine serum, 2 mM glutamine, 10 U/ml penicillin, 100 μg/ml streptomycin, and 250 μg/ml geneticin. Cells were transfected with the expression vectors using Fugene (Roche Diagnostics) and selected with 1 μg/ml puromycin (Sigma). Transfected cells were grown to confluence in HYPERFlasks (Corning), washed twice with PBS, and incubated with serum-free medium for up to 5 weeks with one medium exchange per week.

For purification of His-tagged fibromodulin, the filtered serum-free cell culture supernatant was adjusted to a final concentration of 20 mM Na-HEPES (pH 7.5) and loaded onto a 5-ml HisTrap Excel column (GE Healthcare) using an Äkta Purifier (GE Healthcare). The column was washed with buffer A (20 mM HEPES pH 7.5, 150 mM NaCl, 2 mM CaCl_2_) and the protein was eluted with 500 mM imidazole in buffer A. To remove the His-tag, fractions containing protein were incubated overnight with recombinant His-tagged TEV protease (made in *E. coli* using an expression vector kindly provided by Stephen Curry) while dialysing against buffer A. The reaction mixture was passed over a 5-ml HisTrap Excel column, and the flow-through containing the untagged fibromodulin protein was collected. After concentration using a Vivaspin centrifugal device (Sartorius), the fibromodulin protein was further purified by size exclusion chromatography on a Superdex 75 10/300 GL column (GE Healthcare) using buffer A as the running buffer. The purified protein ran as a single broad band of ~ 55 kDa on reducing SDS-PAGE (calculated: 41.5 kDa + 4 *N*-linked glycans).

The natural affinity of chondroadherin for Ni^2 +^ affinity resin was used to purify the untagged protein. The filtered serum-free cell culture supernatant was adjusted to a final concentration of 20 mM Na-HEPES (pH 7.5) and loaded onto a 5-ml HisTrap Excel column using an Äkta Purifier. Elution was performed with an imidazole gradient in buffer A. The chondroadherin protein was further purified by size exclusion chromatography as described for fibromodulin. Chondroadherin and the collagen-like peptide AB31 were mixed in a 1:2 molar ratio and incubated for 15 min at room temperature. The chondroadherin-AB31 complex was purified by size exclusion chromatography in buffer A.

### Peptide synthesis

Toolkit peptides, as C-terminal amides, were synthesised on TentaGel R-Ram resin using Fmoc chemistry in a CEM Liberty microwave-assisted synthesiser as described [9]. Toolkit peptides have the form: GPC-[GPP]_5_-[27aa]-[GPP]_5_-GPC. A neutral control peptide, GPC-[GPP]_10_-GPC, called GPP10, consists of the flanking regions alone. In all cases, fractions containing homogeneous product were identified by analytical HPLC on an ACEphenyl300 (5 mm) column, characterised by MALDI-TOF mass spectrometry, pooled and freeze-dried. All have been shown, using polarimetry, to adopt triple-helical conformation [50].

### Crystallisation

Crystal screening was done at room temperature by the sitting-drop vapor diffusion method using 96-well plates (Greiner) and a range of commercial screens. A Mosquito nanolitre robot (TTP Labtech) was used to set up 200 nl sitting drops.

Crystals of untagged fibromodulin were obtained from a 10 mg/ml protein solution after one month at condition C6 of the Wizard Classic 3 screen (Rigaku) and at condition G5 of the Index screen (Hampton Research), but these crystals did not diffract to high resolution when cryoprotected with 30% glycerol. A range of crystallisation conditions and cryoprotectants were screened. The best results were obtained by growing the crystals at 26% (w/v) PEG 3350, 200 mM lithium sulphate monohydrate, 100 mM Tris-HCl (pH 8.5) and using an additional 15% PEG 3350 as cryoprotectant.

The chondroadherin-AB31 complex crystallised under a wide range of conditions at acidic pH, but the crystals were later found to contain only chondroadherin. When tested, chondroadherin alone crystallised under the same conditions. The best crystals grew from a 10 mg/ml protein solution in 20 mM Tris-HCl pH 7.5, 150 mM NaCl, 2 mM CaCl_2_ using 200 mM monobasic potassium phosphate (pH 4.8), 20% (w/v) polyethylene glycol 3350 as precipitant. Crystals were harvested in reservoir solution supplemented with 20% ethylene glycol and flash-frozen in liquid nitrogen.

### Crystal structure determination

Diffraction data were collected at 100 K at beamlines I04-1 and I24 of the Diamond Light Source, Oxfordshire, UK. The data were processed using XDS [51] and programs of the CCP4 suite [52] as implemented in the XIA2 pipeline [53]. CC_1/2_ was used to determine the resolution limit [54]. Both fibromodulin and chondroadherin crystals belong to space group *C*2 with two molecules in the asymmetric unit. The phases were determined by molecular replacement using PHASER as implemented in the PHENIX suite [55]. The search models were derived from the crystal structure of decorin (PDB 1XKU) [30]. Automatic and manual building was done using AutoBuild in PHENIX and Coot [56], respectively. Refinement was done using PHENIX. Figures were generated using PyMOL (www.pymol.org).

### Size exclusion chromatography with multi-angle light scattering

Fibromodulin and chondroadherin samples at a concentration of 10 mg/ml were injected onto a Superdex 75 10/30 column (GE Healthcare) connected to a 1260 Infinity HPLC (Agilent Technologies). The running buffer was 150 mM NaCl, 20 mM HEPES (pH 7.5) and the flow rate was 0.1 ml/min. Light scattering and refractive index changes were monitored using in-line Wyatt Mini Dawn and Optilab T-rEX detectors (Wyatt Technology Corp). The data were analysed with the Wyatt ASTRA V software. Each *N*-linked glycosylation site was assumed to add 2 kDa of molecular mass. The extinction coefficients for fibromodulin and chondroadherin were calculated as 32,930 and 40,715 M^− 1^ cm^− 1^, respectively.

### Collagen binding assays

Recombinant purified chondroadherin was biotinylated using biotin EZ-link reagent (ThermoFisher) and dialysed into TBS to remove excess biotinylation reagent. Collagen peptides from Toolkit II and III [57], [58] were coated on a 96-well plate at 10 μg/ml in 20 mM acetic acid overnight at 4 °C. BSA and (GPP)_10_ triple-helical peptides were used as negative controls. The following incubations were conducted at room temperature. Plates were rinsed three times with TBS and blocked for 1 h with 5% BSA in TBS. After rinsing with TBS, plates were incubated with biotinylated chondroadherin at 10 μg/ml in TBST (TBS with 0.1% Tween-20) with 0.1% BSA for 1 h. Plates were washed three times with TBST, and incubated with streptavidin-HRP (Millipore) diluted 1:20,000 in TBST with 0.1% BSA for 1 h. Plates were washed five times with TBST, and the binding was detected with TMB substrate (ThermoFisher) and stopped with 2 M sulphuric acid. The absorbance was read at 450 nm.

For chondroadherin-collagen II binding assays the plates were coated with acid-solubilized collagen II (Sigma) diluted to 10 μg/ml in 20 mM acetic acid. The rest of the assay was carried out as above, but chondroadherin was diluted 1:2 in 15 steps starting from 10 μg/ml. Similar procedures were used in chondroadherin-collagen II inhibition assays, in which Toolkit peptides II-26 or III-8 were used as inhibitors at concentrations of 50, 100, 200, 400 μg/ml pre-incubated for 1 h with 10 μg/ml biotinylated chondroadherin.

### Database references

The coordinates of the fibromodulin and chondroadherin structures have been deposited in the Protein Data Bank under codes 5MX0 and 5MX1, respectively.

The following is the supplementary data related to this article.

Fig. S1 Sequences of the LRRs in (A) fibromodulin and (B) chondroadherin. The colouring scheme of the cap structures is the same as in Fig. 1. The LRR consensus is highlighted in red and the repeat length indicated in the last column. Fig. S1
