# Supplementary material for: Structural and functional analysis of two small leucine-rich repeat proteoglycans, fibromodulin and chondroadherin
Source: Matrix Biol. 2017 Nov;63:106–16. doi: 10.1016/j.matbio.2017.02.002 (PMC5618690; doi:10.1016/j.matbio.2017.02.002)
Supplement: Fig. S1 — Sequences of the LRRs in (A) fibromodulin and (B) chondroadherin. The colouring scheme of the cap structures is the same as in Fig. 1. The LRR consensus is highlighted in red and the repeat length indicated in the last column. [file mmc1.pdf]

A

LRR	Fibromodulin sequence	Length
	ECDCPPNF	
I	PTAMYCDNRNLKYLPFVPSR	20
II	MKYVYFQNNQITSIQEGVFDNATG	24
III	LLWIALHGNQITSDKVGRKVFSKLRH	26
IV	LERLYLDHNNLTRMPGPLPRS	21
V	LRELHLDHNQISRVPNNALEGLEN	24
VI	LTALYLQHNEIQEVGSSMRGLRS	23
VII	LILLDLSYNHLRKVPDGLPSA	21
VIII	LEQLYMEHNNVYTVPDSYFRGAPK	24
IX	LLYVRLSHNSLTNNGLASNTFNSSS	25
X	LLELDLSYNQLQKIPPVNTN	20
XI	LENLYLQGNRINEFSISSFCTVVDVVNFSK	30
XII	LQVLRLDGNEIKRSAMPADAPLCLRLA	27
	SLIEI	

B

LRR	Chondroadherin sequence	Length
	ACPQNCHCHSD	
I	LQHVICDKVGLQKIPKVSEK	20
II	TKLLNLQRNNFPVLAANSFRAMPN	24
III	LVSLHLQHCQIREVAAGAFRGLKQ	24
IV	LIYLYLSHNDIRVLRAGAFDDLTE	24
V	LTYLYLDHNKVTELPRGLLSPLVN	24
VI	LFILQLNNNKIRELRAGAFQGAKD	24
VII	LRWLYLSENALSSLQPGALDDVEN	24
VIII	LAKFHVDRNQLSSYPSAALSKLRV	24
IX	VEELKLSHNPLKSIPDNAFQSFGRY	25
X	LETLWLDNTNLEKFSDGAFLGVTT	24
XI	LKHVHLENNRLNQLPSNFPFDS	22
XII (LRRCT)	LETLALTNNPWKCTCQLRGLRRWLEAKASRPDATCASPA KFKGQHIRDTDAFRSCKFPTKRSKKAGRH	

Figure S1. Sequences of the LRRs in (A) fibromodulin and (B) chondroadherin. The colouring scheme of the cap structures is the same as in **Figure 1.** The LRR consensus is highlighted in red and the repeat length indicated in the last column.
